# ‘After mealtime is before mealtime’ – an ethnographic exploration of nursing home residents’ practices of dealing with meal requirements

**DOI:** 10.1186/s12877-022-03595-2

**Published:** 2022-12-22

**Authors:** Doreen Stöhr, Hanna Mayer, Eva Soom Ammann

**Affiliations:** 1 Institute of Nursing Science, University of Vienna, Alser Straße 23/12, A-1080 Wien, Austria; 2 Department of General Health Studies, Division Nursing Science with focus on Person-Centred Care Research, Karl Landsteiner University of Health Sciences, Dr.-Karl-Dorrek-Straße 30, A-3500 Krems, Austria; 3 Health Department, aR&D Nursing, Bern University of Applied Sciences, Murtenstrasse 10, CH-3008 Bern, Switzerland

**Keywords:** Food, Eating, Mealtime, Ethnography, Institution, Nursing Home, Residents

## Abstract

**Background:**

In nursing homes, food is part of the care provided to residents, causing it to be strictly organised within the institutional framework. Moreover, once food has been integrated into the institutional logic, structural and economic aspects regarding organisation of food and eating may dictate individual and social needs, as a theoretical perspective informed by Goffman’s notion of the ‘total social institution’ suggests. This paper describes nursing home residents’ practices of dealing with meal requirements in two Austrian nursing homes, to understand how food integrates into the daily routine and how the institutional setting influences the social and material arrangement of food.

**Methods:**

An ethnographic design was chosen to gain an in-depth understanding. Two urban nursing homes were studied over 21 months (approx. 800 h of participant observation and ethnographic interviews collected). Data analysis took place iteratively, following Grounded Theory strategies.

**Results:**

As the thick descriptions resulting from this procedure show, observing everyday practices of eating in nursing homes reveals complex dimensions of residents being subject to institutional logics, and also demonstrates that residents develop elaborate strategies to deal with the institutional circumstances.

**Conclusion:**

A better understanding of the resulting tensions between the restrictions of living in a formal organisation and the agencies of residents described, may contribute to better understanding the effects of structural constraints and to designing more flexible processes.

## Background

Eating, as a lifeworld activity involving planning, procuring, preparing, distributing, consuming, and digesting food, occupies a large part of everyday life and serves several functions. Eating has a biological and a social component. The intake of food is necessary for life and survival, providing energy, building up and maintaining the body substance as well as controlling the metabolic process [1]. Eating in itself is a recurring action that eliminates hunger and thus creates a sense of security [2]. In most cultural contexts, eating becomes a social act. Meals structure the day and enable social participation and social control. It is in the community that provisioning, preparation, distribution, and consumption is regulated [3]. This also applies to various subcultural communities, including nursing homes and their residents and employees.

Meals are taken in various community contexts. Daily meals usually take place in rather small communities or even alone. Classically, meals are taken in the (extended) family with different constellations on different occasions. Taking meals in a restaurant implies an extension of the community, but with mostly unknown persons. The nursing home also represents an extended community, where people meet regularly for meals and know each other, which is different from eating in a restaurant. Moreover, collectively eating in a nursing home is everyday life, which distinguishes it from eating in contexts such as weddings, birthdays, and holidays like Christmas.

This paper focuses on the arrangement of meals and eating in the subcultural context of Austrian nursing homes and how their actors perform the act of eating within this institutional framework. This perspective not only includes social situations, but also the personal needs of individual nursing home residents as well as the institutional framework. The practices that make up the food culture are described from a resident perspective as a culture of dealing with institutional restrictions in contexts of eating. The intent is to demonstrate how food intake in the nursing home is institutionally shaped and what this entails for residents in everyday life.

In 2019, approximately 96,000 older persons in Austria (total population: 9 million) were cared for in nursing homes by an approximate total of 36,000 caregivers [4]. Persons usually enter a nursing home at advanced age when their care needs cannot be met by formal or informal home care and support services anymore^1^. Physical and/or cognitive impairment and multi-morbidity are very common, and nursing homes usually provide for various care needs. Thus, resident populations have quite heterogeneous needs and capacities, also with reference to administering food and eating. The food in a nursing home is usually adapted to the nutritional needs (e.g. nutrient composition, texture of the food) of the elderly residents. In addition, they can receive individual support for nutrition-related challenges such as pressure sores in the mouth, swallowing and digestive disorders, motoric impairments that make it difficult or impossible to eat independently. Aging is generally associated with losses. In the context of eating and nutrition, these include loss of optimal body composition [5], loss of optimal oral health [5], loss of optimal sensory function [5, 6], and loss of role function [5]. In addition, as Maierhofer [7] suggests, age-related physiological changes occur, such as altered sensations of chewing, tasting and smelling as well as limited mobility, with which comes lower energy metabolism and a decrease in appetite and thirst.

From a somatic health care perspective, the physiological aging process therefore requires an increased intake of high-quality nutrients to avoid malnutrition [8]. This is a common concern in geriatric long-term care [9], and its avoidance is defined as a measurable quality indicator in nursing homes. Therefore, numerous studies shed light on avoiding malnutrition from various research perspectives. However, studies on social interactions in the context of eating in nursing homes are scarce, although it is known in nutrition psychology that social life and social interactions in general have an impact on eating and food intake [2].

The discrepancy between individual preferences and nutritional needs is worth consideration because, as the above-mentioned state of research reflects, nursing homes primarily provide for medical and nursing needs. In fulfilling this institutional purpose, psycho-social needs may not (or cannot) adequately be taken into account, although there is an increasing emphasis on person-centeredness.

According to Goffman´s seminal work in the 1960s [10], nursing homes are total social institutions in which people are isolated from society upon moving into them. In total social institutions, it becomes difficult to continue to live out one’s habits, which leads to a break with previous roles. The cause of this lies in disculturation (ibid.), i.e., a process of unlearning one’s usual habits, because ‘inmates’ basically follow the expectations that the institution imposes on them.

Thus, when providing comprehensively for residents, institutional goals may take precedence over individual needs. Goffman has drawn attention to institutional control and coercion, a focus which has been widely accepted among health institutions, leading to the redesign of nursing homes. However, traits of the total institution remain inherent to nursing home life, as for example Heinzelmann [11] shows, who proposed the term ‘pseudo-total institution’ for present-day nursing homes, or Koch-Straube [12], using the term ‘moderate total institution’, or Dupuis, Smale & Wiersma [13] with the term ‘closed institutions’ to characterise this. As will be shown, focusing on eating in the nursing home from a perspective inspired by the total social institution literature yields novel insights in health science studies.

## Method

### Design

To gain an in-depth understanding of practices related to food culture in nursing homes, an organisational ethnography study design was used. There are certain social patterns in organisations that open up and at the same time limit possible practices within the organisation. With the help of organisational ethnographic studies, such patterns and their effects on social interactions can be explored [14, 15]. Ethnographic methods have been used previously to explore organisational culture in healthcare [16–20], but such research has up to now not focused on food culture.

The results presented here are part of a larger study on food culture and the institutional organisation of food in nursing homes. They represent a first step in exploring the phenomenon under study and make use of an ethnographic strategy [21]. The aim of ethnography is to study situations that are as ‘natural’ as possible, not to create ‘artificial laboratory situations’ that can be controlled by research [22] – a debate also found in American pragmatism or ‘naturalism’ [23]. Here, this methodological strategy was used to investigate food culture as ‘natural’ implicit knowledge in practice, thus aiming at genuine abductive knowledge gain. In particular, the institutional organisation of food and how social actors can/should/are allowed to influence, control and shape food culture in the nursing home will be examined.

The organisational ethnographic approach was particularly suitable to explore nursing home food culture and associated practices since it combines several research methods. These typically include qualitative interviews and/or focus groups, document analysis, and, as the main survey instrument of ethnography, participant observation [14, 24–27].

In order to understand how food is integrated into the daily routine in the nursing home context and what influence the institutional setting has on the social and material arrangement of food, two nursing homes in an urban area were studied over 21 months from July 2014 to April 2016 (approx. 800 h of data collection).

### Data collection

The organisational ethnographic approach suggests that researchers do not remain distanced observers, but that they immerse themselves into the field to become part of everyday work and life. Immersion into the field in ethnographic research aims at gaining ‘insider knowledge’, so that the phenomenon can ideally be observed via the ‘native point of view’ [28, 29]. With this procedure, the tacit knowledge of social actors in the field [30, 31] - a ‘knowing how’ rather than a ‘knowing that’ [32] - can be revealed, which cannot be attained by inquiry or measurement. In research practice, this implies openness in procedure and time in the field.

In order to make institutional practices associated with food culture in nursing homes visible, the first author used the following data collection procedures: field observations with a participating approach, ad-hoc ethnographic interviews that were more or less rigidly structured, scheduled semi-structured interviews, and the study of patient documentation. Observations were written down in a field diary immediately after the field observations. These observational field notes were the main research data. The interview guides were self-developed, they built on the field notes and were thus created and adapted context-specifically. Scheduled interviews were conducted with residents and staff at a chosen location in the institution. Due to irritations of the interview participants caused by the initially used recording device, the researcher took handwritten notes. Ad-hoc ethnographic interviews were conducted spontaneously with employees, residents and relatives and written down in the field notes. In addition, the patient documentation system was consulted, field notes were taken and integrated into the data corpus. The data were stored in a physical archive, safely secured and only accessible to the first author.

### Data analysis

The analysis was guided by an interpretative paradigm, the epistemology of which implies that the researcher is not independent but interacts with participants. The researcher is thus considered part of the object of inquiry, which needs to be systematically reflected in the process of data collection and analysis [33].

Since the ‘reality’ under study cannot be objectively evaluated and the respective implications of the epistemological position on research practice should be taken into account [34], the analysis of the data took place iteratively following Grounded Theory strategies as described in constructivist grounded theory [35]. Dellwing and Prus [14] propose an iterative method of analysis, using coding techniques in which interpretive closure emerges via the processual, ever-new ordering of piles of codes and lines among them. This strategy was used in the study presented here as follows: The field notes were continuously processed by the first author by coding and relating elements to each other and by writing interpretive memos. In the course of initially pragmatic coding and memo-writing procedures, data collection, field protocol writing and processing itself became increasingly analytical, using reflexivity as a systematic tool of interpretation [14]. Furthermore, a cyclical alternation between ‘going native’, the empirical immersion into the field, and ‘coming home’, the theoretical, contextualizing reflection, was increasingly used as a reflexive analytical tool in the process of interpretation [22, 24, 36]. Based on this iterative process of collecting data, processing fieldnotes, coding and memoing, ‘thick descriptions’ were elaborated by the first author, which were continuously discussed with the third author.

### Ethical considerations

The nursing home managements had given a general informed consent that contained the basic agreement to conduct this study. The employees, residents and relatives were informed verbally, and a handout about the research project was given at initial contact. All residents were informed verbally about the study and the role of the researcher in the field at initial personal contact, and their consent was obtained interactively in the situation (ongoing consent)^2^. The iterative approach and continuing interaction with the participants enabled them to shape their research participation and comment on data collection according to their capabilities^3^. In ethnographic research, the responsibility for ethical good conduct lies with the researchers [22]. They have to judge ad hoc during the interaction and decide whether it is ethically justifiable to do research and collect data, or whether the welfare or protection of the participants outweigh it, in which case the researchers should withdraw from the situation in question^4^.

Nursing home residents may be considered vulnerable groups that need to be protected from research. However, if research focuses on precisely this vulnerability and thus has the potential to generate new knowledge contributing to care provision that is sensitive to such vulnerabilities, the vulnerability of nursing home residents may be reduced through involving such groups in research despite their vulnerability. The ethnographic method is adaptive and therefore adept at accounting for vulnerabilities to conduct research in a sensitive and reflective manner.

## Access to the field and description of field sites

Access to the field sites was mediated through the general management of a facility group operating nine nursing homes in Vienna. Contact was established with the directors of two nursing homes. The two nursing homes lack cooking facilities for reasons of space and cost, but are catered for by an external food supplier that offers the Cook and Chill system^5^. The general conditions of field research were defined and agreed with the directors.

In Facility 1, which houses about 90 residents with various care needs, the first author was allowed to observe for three weeks in July 2015, 7 days a week from about 7.30 a.m. to about 8 p.m. The care unit manager of the ward to which the first author was primarily assigned paid considerable attention to the situations and activities to which the first author was granted access. A plan was prepared that included whom the first author should meet, when to go where, and whom to work with or to interview.

The starting point was a ward to which the first author returned each time after visiting other wards or other residents. The first author was asked to ‘supervise’ and support residents during meals, which enabled her to conduct observations and ethnographic interviews during mealtimes. As a rule, this task is performed by caregivers, but, often, due to time constraints, the task is not well-liked. Since access to the field was very strongly regulated in Facility 1, the role allocated to the participant observer [37] could hardly be negotiated, which meant that it was not possible to establish deeper involvement in everyday activities with the staff. Instead, however, residents actively engaged in settling the first author in quickly and smoothly, and find her way round the field by sharing their everyday knowledge of the cultural rules and conventions that need to be respected on their ward – which prove to be a fruitful source of knowledge for the study.

In Facility 2, where around 250 residents with various care needs are cared for, a 2-week internship was initially planned, but after the positive feedback from residents and especially staff on the presence of the first author, it was extended to one day of internship every two weeks for one and a half years.

After an acclimatisation period, the role in the field was re-negotiated and the first author became increasingly integrated over time. She was allowed to be present during handovers on the ward, to look over the manager’s shoulder during daily work, to share employee breaks without conversations stopping when she entered the room. The nursing staff in particular became more and more relaxed in the first author’s presence and opened up, and some identified her as one of their own, who had to deal with the comforts and difficulties of everyday life on the ward the same way they did. As a result, data could be collected over a long period, which made it possible to have recurring conversations and experience changes occurring over that period. This data could thus be used to explore the nursing home food culture, gain tacit knowledge, and transform it into scientific knowledge through analysis and reflexivity.

## Results: after mealtime is before mealtime

In the following, the results are presented as ‘thick descriptions’ [21] of concrete situations associated to nursing home food culture, which will be theoretically contextualised. All names have been changed to protect the participants´ right to anonymity. The focus of this paper is on the residents of the two nursing homes^6^.

### The fear of unpleasant consequences if rules cannot be followed

It is my (i.e. the first author’s) 2nd day in Facility 1 when Mrs. Miran must wait unusually long before being brought back by an occupational therapist from group therapy in the basement to her ward on the 4th floor. On that day, the therapist, a community service worker, and I have the task of getting all 16 group therapy participants back to their wards. Since only one of three elevators is working, which offers space for a maximum of two wheelchairs and an escort, Mrs. Miran, twelve other residents with wheelchairs or rollators and I are waiting in the basement in front of the lift. Mrs. Miran keeps looking anxiously at her watch and squirming in the seat of her wheelchair. At some point she comments that there are only ten minutes left until lunch. The woman with the rollator nearby, Mrs. Schlink, then looks in her handbag for her watch, which she does not find. The man in the wheelchair next to her tries to relieve the pressure, noting that ‘those on the ward’ know where they are and ‘would not immediately send out a search party’. This is apparently not a convincing argument for the two women, because generally no food is set aside on their ward for late comers, and they would hence have nothing to eat. The man then remarks that missing the meal is not that bad, since lunch is generally inedible, and that he would prefer the afternoon snack anyway. Mrs. Schlink moves with her rollator to the staircase, intending to walk to the 2nd floor so that she will be on time for lunch. Mrs. Miran seems to find this idea persuasive and also moves towards the stairs with her wheelchair. She is already out of the wheelchair when the women realise that they will not be able to carry the rollator or the wheelchair up the stairs. However, Mrs. Schlink continues on her way, announcing that she will ask a ‘strong caregiver’ to fetch her rollator, because she can hold on to the handrail while climbing the stairs and does not need a rollator. My argument that I could run upstairs and quickly let the staff on the wards know they will be late does not persuade them to turn back. Only if I had brought their food downstairs would they have been convinced to abandon their plans, a nurse tells me afterwards. She had discussed the event in detail with Ms. Miran in the following afternoon hours.

After ten minutes of stairclimbing, however, Mrs. Miran feels herself weaken and allows me to help her back into the wheelchair via the 3 steps she had climbed in the meantime. After another ten minutes I can accompany Mrs. Miran into the elevator to go to the 4th floor. She asks me to take her directly to her room, where she usually has her lunch, because she hopes that her lateness has not yet been noticed. Once in her room, she asks me to find out if the food has already been distributed. Shortly after I leave the room, the nursing assistant that is responsible for lunch approaches Mrs. Miran and asks her where she has been and why she was not in her room when the food was distributed. Now there would only be leftovers, which she says can be warmed up in the microwave. However, neither Mrs. Miran nor I are offered the chance to explain her tardiness. Rather, she is left with the onus of being unreliable and uncompliant, and she feels uncomfortable. I also feel guilty about not delivering Mrs. Miran to her room on time. Eventually, we look at each other speechlessly and find no words for the situation. In addition, the therapist who organises the group was held responsible for the fact that, although Mrs. Schlink arrived on the 2nd floor in time for lunch, she was on foot. The therapist was later even blamed for neglecting her supervision duty and told that she was lucky nothing else happened to Mrs. Schlink, apart from having to go to bed at noon because of the exertion.

From my ethnographic participation in this event, I, being a rookie in this setting, learned some implicit rules and was taught that I had better follow them to avoid being sanctioned as well. The rules concerning mealtimes and their modalities are given high priority, and not respecting these rules can be sanctioned in the event of non-compliance - not only by residents, but also by staff members who perform tasks for and with the residents (here, e.g., the therapist). Being late for meals or not appearing in an appropriate manner will be reprimanded. The scene showed very impressively how nursing home culture is shaped and structured by food. Right at the beginning of my fieldwork stay, I experienced what it meant not to follow the implicit rules and, as a newcomer, I inevitably had to develop a guilty conscience. The collective commotion in front of the elevator, the extraordinary measures the two women jointly undertook to manoeuvre their way out of the situation, the restrictions that residents have (here, due to limited mobility), show very clearly the necessity of adhering to the rules. Therefore, the residents always seem to be on their guard. As described above, residents even tend to actively adapt and choose the lesser of two evils – Mrs. Schlinks’ attempt to climb the stairs from the basement to the 2nd floor instead of waiting for the elevator exemplifies this in an almost absurd way.

A fear of unpleasant consequences of institutionally inappropriate behaviour is present for many residents. Besides asking questions about not eating, threatening with infusion or oral nutrition supplements or informing family members have also been observed – potential sanctions related to not following – or not being able to follow – rules associated to eating. The above example, however, also illustrates traits of the total institution with regard to spaces of agency offered to residents with the aim of allowing them autonomy and self-determination, such as activity groups. Nursing homes provide their residents social and recreational activities in which the residents can participate upon their choice. However, these activities offered are not comparable to the things residents used to do themselves. They lose a great deal of control when they enter a nursing home, since they do not have as much freedom and independence as they had before. So they have no choice but to abide by the prevailing rules and regulations of the nursing home and the staff. As part of the latter’s possibilities, attempts are made to create as pleasant an atmosphere as possible, but the prevailing institutional conditions make this much more difficult.

Thus, most residents prefer not to accept the offer of activity programmes, in which participation is voluntary. In contrast, in an ergo- or physiotherapeutic individual hour, residents and the nursing staff accept that participants might be late for lunch because therapy prescribed by the doctor has priority. This illustrates very clearly the dichotomy of somatic nursing and caring in a residential environment.

### Overcoming eating impediments

Adhering to rules was also observable with respect to individual eating behaviours, as exemplified by a scene observed a few days later. In the common room^7^ of Facility 1, those residents who do not take lunch in their room sit at their usual places and wait for the soup. Only the clattering of the dishes can be heard, the residents do not talk. After the table has been set with napkin and cutlery 30 min before and the residents have been given a bib, the soup is put on the table for them. Some residents spoon up the soup on their own, others are helped. A care assistant sits at a table with four residents and assists two residents in eating. Another care assistant sits at a table with six residents and assists three of them in eating. For example, they thicken the soup, guide a resident’s hand holding a spoon to their mouth, remind the residents to continue eating or feed them directly. There is no talking, not even when reminding residents to eat, which is done by touching their arm or hand and by eye contact. At another table there are three residents who do not need any support, eat independently and would be able to express their preferences and needs; however, nobody is talking.

While in the common room everything is quiet, the adjacent corridor is busy, where the nurse and a third care assistant are distributing the food to those residents who eat in their rooms^8^. Out in the corridor, there are short conversations among staff and between staff and residents, although the latter cannot be heard in the corridor; what is said does not leave their rooms.

The ward physician, followed by the ward manager, enters the common room. They go directly to Mr. Holand, who is in his 60s and much younger than the other residents on the ward. After a stroke three years ago, he only communicates by making sounds; his movements are extremely slow. He is therefore dependent on a wheelchair and on someone to assist him eating. At the moment, he sits alone at a table in his wheelchair, without assistance, wearing only an undershirt with his trousers and looking apathetic. The ward physician and the ward manager stand before him and discuss his state of health and his eating habits in public. He has ‘got worse’, they state, since he barely eats anything anymore, even when assisted by a carer. Neither of them can explain why this is suddenly the case. The doctor suspects that it could have been another stroke, so she decides that a blood count is needed. Thereupon, the ward manager sends a nurse to retrieve the necessary utensils for taking the blood sample. The doctor then takes a blood sample from the resident on the spot. She pushes the soup aside to put down her utensils. She says to the resident that they have to take his blood now, stretches his arm, puts the tourniquet around his right upper arm and tightens it. Then she inserts the needle, and the blood runs into the 4 tubes one after the other. She releases the tourniquet on his upper arm, pulls out the needle, puts a plaster on it and leaves, followed by the ward manager. Mr Holand still does not move. He has not even touched his soup. A resident sitting in his immediate vicinity, who has only recently moved into the facility, observes what is happening and then forgets to eat her soup. She is immediately admonished to do so by one of the table caregivers. In response she pushes the bowl of soup away from herself without saying a word. The other residents, by contrast, seem to be little affected by the event. No change in the atmosphere is noticeable at the more distant tables.

Obviously, the situation of residents being disturbed by medical interventions during meals and staff making residents’ personal medical matters public has only peripherally affected the old-established residents, as such things seem to be part of everyday life in the nursing home. But it could also be an indication that they have kept quiet in order not to attract negative attention. Possibly also because not eating could then be interpreted as a deterioration in their state of health [38], and therapeutic consequences could be deduced from this, which the scene described illustrates to everyone. Thus, the residents try to avoid attention to their poor eating by eating in an adapted way.

In the situation described, Mr Holand was (half) publicly made into a patient by having his health condition diagnosed during mealtime. On the one hand, the environment in which this happened is inappropriate, because it was actually about eating. On the other hand, one would assume that a diagnosis of health condition should be comprehensive and take place in a private setting. At this point, the importance of exercising control over food intake in the nursing home became clearly visible to everyone present. It was demonstrated in full clarity that therapeutic consequences are to be expected if food is not eaten. It should also be noted that only the new resident reacted with shock, the other residents either looked away in order not to have to deal emotionally with what was happening or behaved in an adapted manner in order not to attract attention. They adapted their behaviour and their needs to the institutional context. The dichotomy of patient/resident that is inherent in the institution of the nursing home [11] becomes very clear at this moment. On the one hand, there is an attempt to respond to the resident individually, and on the other hand, the resident becomes a patient when the professional actors in the nursing home feel they must intervene in order to fulfil their task properly.

### Organising routines around mealtimes

As has been shown so far, the institutional context thus rigorously structures lunchtime for the residents, through rules about where, when and how to eat, but also through the gaze of somatic nursing care that intersects with and sometimes clearly overrules social and emotional aspects of mealtimes. This centrality of eating in the nursing homes studied is not restricted to lunches, but seems to be of systematic relevance: All the activities in a nursing home day, whether those of the residents or the (nursing) staff, are aligned with the five to six mealtimes.

Routinely, a list is drawn up of who among the nursing staff on a given day is responsible for what tasks in the distribution of meals: breakfast is to be served from around 8 a.m. for all residents, and the fork lunch (a snack comprising fruits, yogurt, pastry) around 10 a.m. for some residents. In their morning break at about 10.30 a.m., staff are reminded who has to deal with lunch at noon. As an implicit rule, 30 min after food has been served, it is cleared away again.

Usually, nursing staff take lunch in the break room after serving the residents food. Those who are still busy feeding residents take their meal a little later. For them, the food is portioned and held by those colleagues who are not ‘busy with eating stuff’ that day. During the staff lunch break, it is negotiated who must distribute the afternoon snack at 2 pm. Staff responsible for this are exempt from distributing supper at 5 pm and can leave earlier. During the lunch break, it is also planned who has to deal with breakfast the next day, so that there are no discussions on that during the morning handover. Serving breakfast is not one of the popular tasks of the day because it is time consuming. Staff are instructed to ask each resident what they would like for breakfast, although that is the same for most residents every morning. There are lists for this, which must only be worked through in the morning. However, it is more time-consuming if breakfast is also timed to coincide with the resident’s awakening or time preferences and must then be coordinated with the colleagues responsible for personal hygiene in the morning. Some residents prefer to have breakfast dressed; others insist on being allowed to have breakfast in bed at a certain time. This attempt to organise meals individually, and thus accommodate residents, requires coordination with both residents and nursing colleagues. In contrast, lunch has to be strictly timed, partly because the food carts are equipped with a timer that allows them to be opened only at 11:30 a.m. The carts are then picked up by an external company at 1 p.m. sharp. This may be why the institution tries not to be institutional at breakfast.

Eating in a nursing home is a tightly organised affair, and meals rigidly structure the daily routine of the people working and living there. Residents are to some extent obliged to come to terms with these structures, but they also develop strategies to deal with constraints through tight institutional rules.

How difficult it can be for residents to structure and organise their day in the institution of the nursing home around mealtimes was also explained by Mrs. Jenner, who lives in Facility 1. She has managed to fit into the institutional context without having to abandon her needs entirely by establishing a strict routine, which is recognised by the staff. As she likes to sleep in, she wants her breakfast to be served last, around 9.30 a.m., in her room. Then she needs until noon to get ready for the day with the help of the nursing staff. She takes her lunch in the common room. Afterwards she retreats to her bed until the snack. After the snack she insists on watching her programme on TV, and after supper she always talks to her daughter on the phone. Finally, after a late-night-snack, usually yoghurt or an apple, she goes to bed. It is extremely important for her to follow her hard-earned routines, which amounts to preserving her autonomy in an institutional context where basically everything is predetermined. These strict everyday routines of Mrs. Jenner’s make it impossible, as the nursing staff explained, to put Mrs. Jenner in a group, even though she is ‘still in such a good mood’ that she is one of the few residents physically fit enough to participate. Mrs. Jenner explained to me that she had neither time for the ‘lessons’ nor to chat with me, although she would have liked to tell me about her ‘wonderful’ grandchildren. It took her a while to arrange her day around the mealtimes in such a way that the nursing staff would also agree to it. The most difficult thing for her was not being able to set mealtimes according to her needs. The ‘solution’ that she has found is not yet perfect, but she can live with it and come to terms with the structural constraints by strictly focusing on organising her personal routine around mealtimes. It seems paradoxical that she fights for autonomy from the structural guidelines by setting strict rules herself to make other things possible.

### Playing by the institutional rules

The strict timing and the frequency of food servings is also reflected in residents’ comments on how they experience food and meals in the nursing home. Mrs. Miran explained to me that she would be asked many questions if she did not eat her lunch to the satisfaction of the nurses: You didn’t like it again? Why didn’t you eat anything again? Would you like to eat something else? Mrs Miran’s remarks are confirmed by my observations of daily routines: Nursing staff are always vigilant and attentive to the amount of food eaten, as they must document this in the resident’s file. Their primary task in this context is to ensure the provision of somatic nursing care. With regard to food, this includes ensuring nutrition, monitoring the state of health, detecting and assessing irregularities to prevent emergencies or taking appropriate and timely measures. And all this must be properly documented to ensure nursing process, safety, quality, and reimbursement of costs. Furthermore, staff also endeavour to promote the well-being of the residents, which they often strive to achieve through food. Thus, carers keep a close eye on the eating behaviours of the residents. Both the nursing and the caring gaze notice any deviation from the ‘normal’ and investigate it by means of questions. Residents seem not to be comfortable with this watchful gaze.

To avoid questions from caregivers and thus regain more control over their own food intake, residents have developed various strategies. If lunch is not at all tasty, residents switch to having a snack between meals. A late breakfast is also an excuse not to have lunch. But, as some residents explained, sweet snacks are not a good substitute for lunch. Therefore, in Facility 1, bread with sausage or cheese was also offered as an afternoon snack.

Mrs Kainz increasingly opts for snacks between meals, as she can never be sure what lunch will be like. However, if lunch was not to her liking and she let it go back almost untouched, the comments of the nursing staff would be unpleasant. In addition, she would have to wait until there was something to eat again, because in principle she very much likes to eat. Then, she remarked, one could ‘keep one’s head above water’ with the snack at 2 pm, but the sweet pastries were not a smart alternative, because for her only savoury dishes are considered a ‘full meal’. This could also be a slice of bread with sausage or cheese. In Facility 2, where she lives, however, there is only a sweet pastry for snacks and not bread with a savoury topping. As Mrs Kainz does not have visitors, she cannot organise any alternatives to the foreseen meal plan and the selection of additional meals in the facility if she does not like what is offered, as is the strategy of Mr Armbauer, for example. His daughter comes to visit him once a week and brings all the food that he ‘misses’ in Facility 1. Besides home-cooked soups, which are warmed up for supper for Mr Armbauer – if the personnel are willing to do so –, family baked goods, puddings, yogurts, juices and sausages are brought by his daughter. These are kept in the ward fridge, labelled with the resident’s name, and are handed to Mr Armbauer on request – again, if the staff is willing to do so. Because he is looking forward to these special treats, he makes sure that he leaves ‘enough space’ for it during the day’s mealtimes. Therefore, he does not take a snack in the afternoon and only eats enough of lunch so that no questions are asked.

Another resident strategy to escape the gaze and potential questions of carers is to eat in one’s own private room. Mrs. Schiefer and Mrs. Horacek share a room in Facility 2 and have succeeded in getting permission to eat meals together in their room, allowing them to avoid gazes and constant caregiver questioning as to how much was eaten or not eaten and why. Mrs. Schiefer is diabetic, obese and suffers from an intestinal disease. She is therefore given a light diet, which she is not keen on. Her roommate Mrs. Horacek gets an enriched diet because she is a ‘poor eater’, as the carers point out. Mrs. Horacek was therefore often asked by the carer why she had left food on her plate. In reaction to this, she started to ‘make the food disappear’ but was ‘caught’ by the carers when she put it in her nightstand. To get Mrs Horacek out of this embarrassing and awkward situation, Mrs. Schiefer offered to eat her food. Mrs. Schiefer greatly enjoys being able to eat ‘good things’ again and to feel satisfied. Sometimes neither of them likes the lunch; then Mrs. Schiefer disposes of it in the toilet, because she is more mobile. In this case, Mrs. Horacek fetches a snack in the afternoon, which is then eaten by Mrs. Schiefer, because she is still hungry after the unsatisfactory lunch.

I was also able to observe how two residents swapped individual food components in the common room during lunch when the nursing staff were busy with other residents. One resident took the meat and the other the vegetables. When I asked the two residents about my observation later in the afternoon, they were embarrassed at first and asked me not to pass my observation on to the nursing staff, because they have developed this strategy to let as little food as possible go back and thus avoid questions. This strategy allows them to comply with the institutional rules, but still not completely abandon their preferences.

### Beating the institution at its own game

Getting attention by not adhering to mealtime rules, however, may not only be avoided, but may also be used strategically to one’s advantage. Due to the rigid routines at mealtimes, the emotional attention at this situation often comes up short for the residents.

Mrs. Bauer, who is visited by her family only on her birthday and at Christmas, said that she sometimes felt forgotten. One day she told me about a resident whose name she did not want to reveal. This resident thought it would be wonderful if nurse Sonja had time for her alone. But since time for emotional attention from carers is scarce, the resident in question, after careful observation, found the solution for herself. She simply refused to eat, so that a carer – ideally nurse Sonja – would focus on her, because that is what carers ‘have to do’ and cannot ‘pretend’ they have a more important task. When I guessed that she was talking about herself, she reacted with a negative hand movement and smiled. Mrs. Bauer’s story clearly shows the extent to which residents feel lonely and need attention; but it also shows how they creatively deal with the nursing home culture and its constricting structures, for which they develop their own strategies.

The story above shows that if residents do not have any relatives or do not receive sufficient attention from them carers dedicate more time to those residents. Thus, it seems that carers invest in residents who receive hardly any visitors through their appreciative attitude, which is reciprocated by the resident with affection or docility. These informal exchange relationships involve an individualised give and take between carers and residents, which creates a sense of community [39, 40].

### Striving for autonomy and privacy

As has been shown, residents work out strategies to cope with restrictions in their everyday life and to influence their scope of action for themselves. Thus, they have the chance to arrange their lives within a framework of restricted structures and capabilities in order to maintain or enlarge their autonomy, in a space where they do not have to justify themselves for their behaviours or preferences.

Eating meals in a private room, for example, gives the residents control, because the caregivers are ‘guests’ and for once not the controlling authority. Thus, the meal situation in a private room can be arranged by the residents themselves, at least to a minor extent, and is not orchestrated and monitored by the carers as it is in public. Mrs. Calek’s strategy illustrates this.

While I am handing out the snack at 2 p.m. in Facility 1 and bring Mrs. Calek a lukewarm coffee with lots of milk and a brioche croissant, she talks about her great success at being allowed to eat in her room and says that she does not need anything more for the day. She tells me she finds it very difficult to eat due to her impaired physical mobility, but she does not want to ask the nursing staff for help, not wanting to make them any busier. And so, she feels very uncomfortable about her inability to carry out this recurring activity to her satisfaction. Since she feels so embarrassed about needing help when eating, she has had to ‘fight’ for many weeks to be allowed to eat in her room. At each meal in the common room, she had been reminded by comparing herself with other residents that she could no longer ‘do it’ the way she should. Therefore, she prefers to eat on her own and very slowly, which means that she ingests less and less food, since after about 30 min the food is cleared away to ensure the general flow of tasks and timings. She would never admit that she needs a little more time to eat, and therefore stops eating when her dishes are cleared away. She does not want to be ‘one of the other residents’ who are so ‘miserable’, because she thinks that she does not yet belong in a nursing home. To avoid becoming ‘miserable’, she is taking gymnastics classes to keep fit. Mrs. Calek thus not only uses the institutional offers to prevent deterioration, but furthermore has managed to create a little privacy to compensate for her emerging deficits. However, it is hardly possible to offer her even more comfort within the timeframe, although she would benefit greatly if the food were not cleared away after 30 min. On the other hand, it is through the institutional context that she has the opportunity to maintain and develop her remaining competencies and skills during gymnastics lessons.

On the one hand, Mrs. Calek stays in her room at mealtimes to hide her deficits, because it is important to her to distinguish herself from the other residents who have, in her perception, a poorer general condition [38]. With this strategy, she also tries to escape the vigilant gaze of the carers and its consequences, and also to minimise the danger of co-residents pointing out to the nursing staff that Mrs. Calek is no longer acceptable because she cannot eat autonomously in the collective meal context.

Like Mrs. Calek, some residents tend to group their fellow residents into categories, often distinguishing between three groups characterised as follows: (1) those who are still quite independent and can act autonomously within the institution; (2) those who need more help but are not yet fully dependent; (3) those who are fully dependent, mostly due to their physical condition, and no longer negotiating on autonomy. On the other hand, strategies for hiding deficiencies, as practiced by Mrs. Calek, also entail that due to a lack of knowledge by the staff, no consideration can be given to associated needs. Fitting into the social context, and thus belonging to the community, is essential for Mrs. Calek, because only if she can eat in a cultivated way without spilling and stick to the time allowed for eating meals, will she not be excluded from the community. Thus, she decided to withdraw from the community as a precautionary measure. She took this step ‘voluntarily’ in order not to be publicly excluded and not to have to endure the feeling of rejection.

Autonomy can also be strived for if food from ‘outside’ the ward is consumed, whether it is brought by relatives or self-organised from the snack bar or mini supermarket in the facility or in the immediate vicinity by mobile residents, with the staff’s agreement. However, no help can be offered to them for this; they must be able to manage it completely independently. Mr Friedrich, for example, has negotiated with the nursing staff that every Wednesday and whenever he does not like the offered lunch, he goes to the nearby snack bar outside and buys a kebab, which he pays for out-of-pocket. As the nursing staff remarked, since this arrangement was made, Mr Friedrich has been much more balanced, more satisfied, and only in specific circumstances has he become quick-tempered. Like Mr Friedrich, some residents try to form alliances with employees, which nevertheless depend on their goodwill. Sometimes they invest in building a closer relationship with a specific caregiver, who is then usually more tolerant about sidestepping the rules and not asking questions. The resident’s behaviour, which is to some extent deviant, is then tolerated.

As a rule, eating together in a ‘family’ environment – small to medium-sized groups around a table, usually the ward or residential group – is considered to be the norm in nursing homes [8, 41–43]. Eating in a central restaurant or cafeteria – as Mr Friedrich practices it – is an alternative option that signals freedom, but also requires a certain prosperity because residents have to pay extra for it. This option is, however, usually only chosen by residents if a visitor is announced for lunchtime.

In principle, the residents always look forward to visitors and want to make the visitor as comfortable as possible within the possibilities they have. At this point, the role of host, which can no longer be exercised by residents in the nursing home, appears to be a felt limitation to autonomy. As Gouldner [44] already pointed out, residents have no equivalent form of nurturing social relationships and expressing gratitude in the sense of the ‘reciprocity norm‘.

Furthermore, conclusions can be drawn by the site where food is served to certain residents, which can be understood as a cultural imprint of the institution on individual residents – in contrast to the negotiated small space of autonomy expressed in Mrs. Calek’s story above. Eating in one’s room is, if prompted by the staff, usually more of a ‘banishment’. Either because the resident does not eat properly enough in the group, disturbs the others, is not socially acceptable or wants to be alone because (s)he feels disturbed by the others. But it can also be an expression of weakness or need for help if (s)he does not eat in a ‘normal way’, does not eat at all or needs special assistance with eating.

## Discussion: institutional food culture and practices

In this paper, we have aimed to describe the nursing home culture and practices associated with food from a residents’ perspective, as observed in two Austrian nursing homes. Eating is a normality, an everyday occurrence. But living in a formal organisation changes cultural practices around food and eating. In a nursing home, the intake of meals takes place in a semi-public space, as meals are supervised by staff who are responsible for ensuring, on one hand, that sufficient nutrients are consumed and that deteriorations in individual residents’ health are detected in time, and on the other, that institutional procedures are adhered to. In this context, social control and striving for autonomy are central elements of food culture and practices in nursing homes – elements that resonate with Goffman’s [10] description of total social institutions. In the study presented above, it has become apparent that focusing on food and meals through ethnographic participant observation allows describing systematic traits of nursing home culture from various aspects, particularly with respect to individual residents’ perspectives and the institutional setting. By using an ethnographic strategy, social control and striving for autonomy could be shown to be central elements of food culture and practices. By focussing on the analysis of everyday practices in the institutional setting, it was possible to empirically explore this multidimensionality, taking into account the individual perspectives of the residents.

The results show that living in a formal organisation is mastered by residents with adaptation strategies. Loss of autonomy is often the main reason for moving into a nursing home. Thus, integrating into a total social institution is above all about the loss of identity, which is already exemplified at the time of moving in with the extremely restricted opportunity to bring personal belongings. According to Goffman [10], then, loss of the right to control one’s own time and to determine one’s own daily routine, inability to leave the place or loss of one’s private sphere, as in the case of Mr. Holand, also contribute significantly to the loss of identity. Nevertheless, in total social institutions, residents also develop individual and collective strategies to protect their identity from being erased by the institution. Through eating and drinking, it is possible for residents to preserve and express their identity [45]. However, this opportunity to express their needs and individuality through eating is made more difficult by the standardisation and collectivisation of the processes in the nursing home. This can even lead to conflicts between the nursing staff and the residents when the latter become rebellious and insist on affirming their individuality [46].

To avoid overt conflicts, residents develop different types of strategies, as has been shown above. Some residents obediently follow the rules, as Mrs. Schlink, who walks up the stairs to her room alone for fear of the possible reprisals for being late for lunch, who then, unlike Mrs. Miran, was not berated. Mr Armbauer’s compliance with the rules is also rewarded by the fact that the nursing staff heat up his daughter’s cooked soup for him. A different strategy is fraternisation, i.e. residents helping each other and forming cliques, as practised by Mrs. Schiefer and Mrs. Horacek, who made a pact to avoid food-related questions from carers. Another strategy is to maintain a minimum of freedom and identity by creating an alternative, informal order, the “underlife“ [10], which allows for practices not encouraged by the formal structure of the organisation. Mrs. Jenner is an example of this, who managed to organise her day according to her own ideas, despite the time restrictions, or Mr. Friedrich, who created the freedom even to leave the nursing home for the alternative procurement of food. The strategy of Mrs. Calek, who tries to hide her deficits by any means, i.e., does “stigma management” [47], also clearly shows the residents’ deep desire for normality. All these strategies may be seen as efforts to create personal sense of everyday life in order to maintain identity. They are, therefore, as we state, vital elements of what comprises nursing home culture.

The culture in a formal organisation is socially constructed, a product of groups, not individuals, and based on shared experiences. Still, the exploration of nursing home food culture during fieldwork in the two facilities has shown that the culture in these facilities is quite uniform. In a nursing home, certain rules are set, norms are lived, and concepts are developed that significantly influence everyday life in the institution. These are partly controlled by infrastructures and economic considerations, as visible in the food trolleys, but also by politics and the legal framework.

As the last century has come to an end, the global movement for culture change in nursing homes has grown stronger, and with it the growing awareness that a broader, fundamental change in treatment and care [48, 49] – towards person-centredness [50]– is needed. Despite this, nursing homes are still affected by a lack of home-like models and insufficient staffing [51, 52]. To ensure a person-centred practice, the nursing home as a whole and the professionals in particular must create desirable conditions and experiences for residents. Mayer et al. [53] developed a concept of six fundamental principles of care to guide decision making and actions of professionals which enables residents to do the following: participating in social life and current events; having meaningful relationships; experiencing meaning in everyday life; being free in decisions; maintaining identity and self-esteem; living in a familiar, resident-friendly and home-like environment. However, implementing a person-centred approach is difficult as long as the culture in nursing homes is more institution-centred than individual-centred. On the caregivers’ level, in a setting where people who are highly fragile and dependent are cared for and the focus is increasingly on their somatic needs the holistic caring approach often falls behind.

The results presented in this paper show that, in the context of evolving claims for person-centredness in nursing home care, a further development has taken place on the relationship level between residents and nursing staff. The nursing staff’s fundamental responsiveness to the residents’ needs is very clearly visible. Caregivers focus not only on the individual resident but on the whole situation – as illustrated by Mr Friedrich, whose outbursts of rage, which frightened the other residents and meant extra work for the staff, have subsided because he is allowed to buy his own kebab. Fjær & Vabø [54] describe this staff practice as ‘manipulating’ the social situation by influencing the physical environment, the group composition, the timing of events as well as staff’s presence/absence, which they appraised as ‘good’ nursing practice. Thus, one may say that current practices in nursing homes do not only focus on individual persons, as the notion of ‘person-centredness’ suggests, but also on situations and the active shaping – or: designing – of both social and material dimensions.

Still, despite such attempts to establish a person-centred practice that seems to be aware of social-material contexts of care, the results of our study clearly show that such practices are limited by the structuring force that institutional constraints pose on the organisation of everyday life around food. Yet, our results also illustrate that there is a certain amount of scope left for how food can be arranged and how mealtimes can be organised in the institution – i.e., there is potential to design the food culture in a given nursing home, even within structural constraints and institutional requirements. Culture is inherent – something implicit, something that has developed, something ‘quasi-natural’ – but not something immutable. Responsibility for such organisational cultures lies with the organisation. Thus, nursing homes can organise, adapt and hone this.

The institutional character of the nursing home restricts older people’s decision-making. The challenge in nursing home care is to balance the tension between individual needs and the collective dimensions of care [55]. Or, to put it differently, handling the balance between institutional requirements and individual needs of the residents. At this point, our results have shown how staff who are significantly involved in shaping the nursing home (food) culture are called upon to interact if the practices described in this paper should be adjusted or changed.

Professionals interact and thus participate in the cultural shaping of the practices. From this it can be concluded that they are also the ones who could be agents of change. The ethnographic descriptions in this paper illustrate that staff already does so on a small scale - by e.g. accommodating special requests and helping residents to use their scope of action. For a sustainable change of culture, however, more than person-centred moments in the form of situational support are needed, and it will also need more than additional interventions associated to food organization or intake. As a first step to initiate and implement sustainable development and innovation – or, in other words, a cultural change – the authors suggest that the existing culture in its different facets has to be known and understood (see also [55]).

According to current conceptions of person-centred care, one strategy to strive for cultural change in a nursing home is to put a central focus on the implementation of person-centred care: As the body of literature on how to implement person-centred care in healthcare organisations [56, 57] states, cultural change oriented towards person-centred care is needed and would also allow to design eating in a person-centred way. For this, a practice development would be needed, the goal of which would be to develop a person-centred culture [56]. As McCormack and colleagues [56] state, this cannot be done on the initiative of individual persons or wards, but needs to involve the organisation as a whole, in order to allow for staff to be able to act in a person-centred way in the micro-interventions as well. Thus, in the light of the results of our study, it can be stated that staff being aware of nursing home (food) culture and using available scopes of action may not be enough to change nursing home culture. It also takes the commitment of the institution to embrace change through collective reflection on its practices.

Based on our results, one can intuitively see how deeply embedded the culture is and may conclude that changing the entire nursing home culture is a complex and difficult endeavour as current practices are so deeply rooted in ‘quasi-naturalness’. It is, however, precisely this ‘quasi-naturalness’ that has to be reflexively questioned, it has to be understood how it influences one`s own practice in order to effect change. Our results also indicate that ideals of ‘good care’ are challenging to realise, both for organisations and staff. Therefore, an examination of the institutional and professional design possibilities – i.e. the (more or less) deliberate shaping of practices and environments [54] – may contribute to bringing about change within the frame of cultural ‘quasi-naturalness’. By illustrating through ethnographic ‘thick description’ how nursing home food culture is shaped and how residents deal with institutional constraints, we intend to contribute to this change.

In order to gain a deeper understanding of how food culture in Austrian nursing homes may be changed, the study of everyday food practices presented in this paper was complemented by a study focusing on networks and processes of food organisation in order to better understand how nursing home food culture in Austrian nursing homes is organisationally and strucutrally shaped by responsible professionals, in order to identify opportunities to re-design environments and practices aimed at cultural change (corresponding publications are in preparation).

## Conclusion

This also puts to the fore how those working and living in nursing home organisations are both dependent on structural constraints. But they are not incapable of action; they actively participate and contribute. Following from this, the organisation of food and meals is part of the institutional set-up of a nursing home – but can also be negotiated and dealt with by residents and staff. And organisations may, for example, contribute to culture change by investing in reflecting on implicit effects of nursing home culture and in designing structural frameworks that facilitate the agency of staff and residents within transparently defined boundaries. In other words, there are opportunities for organisations and professionals to change nursing home culture by strengthening negotiation and situational deviation from rules.

## Data Availability

The data are not publicly available due to containing information that could compromise research participant privacy.

## Footnotes

1 In principle, residents of a nursing home in Austria must pay for the costs of care. If their personal income is not sufficient, the costs are covered by the public authorities. Since 2018, it is no longer permissible to draw on the assets of nursing home residents or their relatives and heirs to cover nursing care costs. In order to obtain a place in a nursing home, there must be an actual need for care (in some federal states, care allowance level 4), and care elsewhere - for example by relatives or mobile services - must no longer be possible or reasonable. Waiting times for a place in a nursing home vary from a few days to several months, depending on the location and care requirements.

2 In case a resident was under legal guardianship, informed consent was obtained from the legal guardian at the beginning of the study. In case of scheduled interviews (residents, relatives and staff), a declaration of consent was previously signed by participants.

3 Since participant observation was the main data generating tool and the first author engaged in interactions with residents in ways that were adaptive to their physical, mental and communicative capabilities and their willingness to interact, there were no systematic resident capability assessments done by the first author before engaging in observations or ad-hoc conversations.

4 During ethnographic research, there may also arise situations in which the researcher may feel ethically obliged to react to e.g. perceived misconduct of professionals. In the case of the study presented here, observed situations were not immediately threatening to the involved participants and could be discussed in ad-hoc conversations. If major ethical challenges would have arisen, the first author would have been able to contact a pre-defined person in each institution to address the issue and to strive for a solution compatible with the institutional rules and regulations.

5 The food is prepared in a central kitchen. The vacuum-packed food is quickly cooled down (sous vide) in a metal trolley and then delivered in a cooled state to the individual buildings, where the next day the food is automatically heated and opened on the wards 45 min before it is served to the residents on the wards using a ladle system.

6 Further publications will focus on the staff and organisation.

7 The common room is mainly used as a dining room. However, celebrations are also held in it. In addition, some residents spend their day in the common room after being brought there by the staff and usually do not leave it until they are brought back to their room at the end of the day.

8 Those who have negotiated this with the staff are allowed to eat in their room. Those who need special assistance with eating have their meals in the common room, because it is then less strenuous for the carers and they have a better view of the residents.
